# Efficacy and Safety of a Tetravalent Dengue Vaccine (TAK-003) in Children With Prior Japanese Encephalitis or Yellow Fever Vaccination

**DOI:** 10.1093/infdis/jiae222

**Published:** 2024-04-29

**Authors:** Chukiat Sirivichayakul, Shibadas Biswal, Xavier Saez-Llorens, Eduardo López-Medina, Charissa Borja-Tabora, Lulu Bravo, Pope Kosalaraksa, Maria Theresa Alera, Humberto Reynales, Luis Rivera, Veerachai Watanaveeradej, Delia Yu, Felix Espinoza, Reynaldo Dietze, LakKumar Fernando, V Pujitha Wickramasinghe, Edson Duarte Moreira, Asvini D Fernando, Dulanie Gunasekera, Kleber Luz, Rivaldo Venâncio da Cunha, Ana Lucia Oliveira, Martina Rauscher, Huihao Fan, Astrid Borkowski, Ian Escudero, Suely Tuboi, Eric Lloyd, Vianney Tricou, Nicolas Folschweiller, Inge LeFevre, Luis Martinez Vargas, Derek Wallace, for the TIDES Study Group, Asvini Fernando, Charissa Borja-Tabora, Chukiat Sirivichayakul, Delia Yu, Dulanie Gunasekera, Eduardo López-Medina, Edith Johanna Rodriguez-Arenales, Edson Duarte Moreira, Felix Espinoza, Hector Velásquez, Humberto Reynales, Kleber Luz, Jose Jimeno, LakKumar Fernando, Lulu Bravo, Luis Martinez Vargas, Luis Rivera, Maria Theresa Alera, Onanong Manacharoen, Pio Lopez, Pope Kosalaraksa, V Pujitha Wickramasinghe, Reynaldo Dietze, Rivaldo Venâncio da Cunha, Veerachai Watanaveeradej, Xavier Saez-Llorens, Manja Brose, Shibadas Biswal, Yanee Hutagalung, Suely Tuboi

**Affiliations:** Department of Tropical Pediatrics, Faculty of Tropical Medicine, Mahidol University, Bangkok, Thailand; Takeda Vaccines, Inc., Cambridge, Massachusetts, USA; Pediatric Infectious Diseases, Hospital del Niño Dr José Renán Esquivel, Sistema Nacional de Investigación at SENACYT, Centro de Vacunación Internacional (Cevaxin), Panama City, Panama; Centro de Estudios en Infectologia Pediátrica, Universidad del Valle and Clínica Imbanaco Grupo Quironsalud, Cali, Colombia; Clinical Research Division, Research Institute for Tropical Medicine, Muntinlupa, Philippines; Pediatrics, University of the Philippines Manila, Ermita, Philippines; Faculty of Medicine, Khon Kaen University, Khon Kaen, Thailand; Virology, Philippines– Armed Forces Research Institute of Medical Sciences Virology Research Unit, Cebu, Philippines; Clinical Research, Centro de Atención e Investigación Médica, Bogotá, Colombia; Hospital Maternidad Nuestra Senora de Altagracia, Santo Domingo, Dominican Republic; Department of Pediatrics, Phramongkutklao Hospital and Kasetsart University, Bangkok, Thailand; Pediatrics, De La Salle Health Sciences Institute, Dasmariñas, Philippines; National Autonomous University of Nicaragua, León, Nicaragua; Núcleo de Doenças Infecciosas, Centro de Ciências da Saúde, Universidade Federal do Espirito Santo, Vitória, Brazil; Centre for Clinical Management of Dengue and Dengue Haemorrhagic Fever, Negombo General Hospital, Negombo, Sri Lanka; Paediatrics, University of Colombo, Colombo, Sri Lanka; Laboratory of Molecular Epidemiology and Biostatistics, Associação Obras Sociais Irmã Dulce Hospital Santo Antônio and Oswaldo Cruz Foundation, Bahia, Brazil; Faculty of Medicine, University of Kelaniya, Colombo, Sri Lanka; Faculty of Medical Sciences, University of Sri Jayawardenenpura, Nugegoda, Sri Lanka; Instituto de Medicina Tropical da Universidade Federal do Rio Grande do Norte, Natal, Brazil; Department of Infectious Diseases, Universidade Federal de Mato Grosso do Sul, Campo Grande, Brazil; Department of Infectious Diseases, Universidade Federal de Mato Grosso do Sul, Campo Grande, Brazil; Takeda Pharmaceuticals International AG, Zurich, Switzerland; Clinchoice Inc, Fort Washington, Pennsylvania; Takeda Pharmaceuticals International AG, Zurich, Switzerland; Takeda Vaccines, Inc., Cambridge, Massachusetts, USA; Takeda Distribuidora Ltda, Sao Paulo, Brazil; Takeda Vaccines, Inc., Cambridge, Massachusetts, USA; Takeda Pharmaceuticals International AG, Zurich, Switzerland; Takeda Pharmaceuticals International AG, Zurich, Switzerland; Takeda Pharmaceuticals International AG, Zurich, Switzerland; Centro de Atención e Investigación Médica, Santo Domingo, Dominican Republic; Takeda Vaccines, Inc., Cambridge, Massachusetts, USA

**Keywords:** dengue, Japanese encephalitis, yellow fever, vaccination, TAK-003 effectiveness

## Abstract

**Background:**

We explored the impact of prior yellow fever (YF) or Japanese encephalitis (JE) vaccination on the efficacy of Takeda's dengue vaccine candidate, TAK-003.

**Methods:**

Children 4–16 years of age were randomized 2:1 to receive TAK-003 or placebo and were under active febrile surveillance. Symptomatic dengue was confirmed by serotype-specific reverse-transcription polymerase chain reaction. YF and JE vaccination history was recorded.

**Results:**

Of the 20 071 children who received TAK-003 or placebo, 21.1% had a YF and 23.9% had a JE vaccination history at randomization. Fifty-seven months after vaccination, vaccine efficacy (95% confidence interval) was 55.7% (39.7%–67.5%) in those with YF vaccination, 77.8% (70.8%–83.1%) for JE vaccination, and 53.5% (45.4%–60.4%) for no prior YF/JE vaccination. Regional differences in serotype distribution confound these results. The apparent higher vaccine efficacy in the JE vaccination subgroup could be largely explained by serotype-specific efficacy of TAK-003. Within 28 days of any vaccination, the proportions of participants with serious adverse events in the YF/JE prior vaccination population were comparable between the TAK-003 and placebo groups.

**Conclusions:**

The available data do not suggest a clinically relevant impact of prior JE or YF vaccination on TAK-003 performance. Overall, TAK-003 was well-tolerated and efficacious in different epidemiological settings.

***Clinical Trials Registration.*** NCT02747927.

## Teaser

A considerable proportion of participants had prior yellow fever or Japanese encephalitis vaccination in the ongoing 8-country pivotal efficacy trial of Takeda's dengue vaccine candidate. Regardless of these previous vaccinations, TAK-003 was well-tolerated and efficacious in different epidemiological settings.

Flaviviruses transmitted by arthropods such as mosquitoes are estimated to infect nearly 400 million people yearly, with clinical manifestations ranging from mild illness to severe and life-threatening disease [1]. Recent decades have experienced a global spread of flavivirus diseases, which are often associated with recurrent, explosive epidemics [1]. Dengue alone is responsible for approximately 96 million symptomatic infections annually and is endemic in >100 countries in the World Health Organization regions of Africa, the Americas, the Eastern Mediterranean, South-East Asia, and the Western Pacific, with periodic outbreaks [2, 3]. Vaccines are available and widely used against some flavivirus diseases in parts of the world, such as yellow fever (YF), Japanese encephalitis (JE), and tick-borne encephalitis [4]. In the meantime, considerable progress has been made in dengue vaccine development, raising the possibility that dengue could become a vaccine-preventable disease.

A live-attenuated tetravalent dengue virus (DENV) vaccine has been developed by Takeda (TAK-003). It is comprised of a DENV-2 backbone and 3 chimeric viruses with pre-membrane and envelope protein genes from the DENV-1, DENV-3, and DENV-4 strains [5]. The vaccine is licensed for use in Argentina, Brazil, Colombia, Indonesia, Malaysia, Thailand, the European Union, the European Economic Area, and the United Kingdom, and is under review by regulatory authorities in several countries. Clinical trials have shown that TAK-003 is well-tolerated and immunogenic in healthy adults living in dengue-nonendemic regions, and in adults and children living in dengue-endemic regions when given as 2 subcutaneous injections 3 months apart [6–17]. In the ongoing pivotal phase 3 efficacy trial (ClinicalTrials.gov NCT02747927) in Asia and Latin America, 20 071 participants aged 4–16 years received the first dose of vaccine or placebo between September 2016 and March 2017 [6, 8, 17].

The overall vaccine efficacy (VE) against virologically confirmed dengue (VCD) caused by any serotype from 30 days after the second dose to the end of year 1 was 80.2% (95% confidence interval [CI], 73.3%–85.3%) [8]. The overall VE against hospitalized dengue from 30 days after the second dose to the end of 18 months was 90.4% (95% CI, 82.6%–94.7%) [6]. By 4.5 years postvaccination, cumulative VE (95% CI) from first dose against VCD was 61.2% (56.0%–65.8%): 53.5% (41.6%–62.9%) in participants who were seronegative against all 4 dengue serotypes at baseline and 64.2% (58.4%–69.2%) in participants who were baseline seropositive for any dengue serotype [18]. VE (95% CI) against hospitalized VCD was 84.1% (77.8%–88.6%) during that period: 79.3% (63.5%–88.2%) in baseline seronegative participants and 85.9% (78.7%–90.7%) in baseline seropositive participants. VE varied by serotype; exploratory evaluation has suggested that the vaccine is efficacious against DENV-1 and DENV-2 regardless of baseline serostatus, whereas VE against DENV-3 and DENV-4 was only demonstrated in baseline seropositive participants. In baseline seronegative participants, the available data did not suggest efficacy against DENV-3, and the low number of DENV-4 cases precluded a robust assessment of VE [18].

The potential effect of prior vaccination against JE or YF on dengue vaccine performance is of interest because cross-reactive antibodies from previous flavivirus infection(s) may alter the immune response to subsequent flavivirus vaccines [19], and exposure to a prior flavivirus may increase the risk of developing severe dengue [20]. About 45% of trial participants had a history of JE and/or YF vaccination, and an interim analysis 1.5 years after vaccination showed a trend of increased VE in participants previously immunized against JE [6]. However, the data were influenced by a relatively high proportion of DENV-1 and DENV-2 cases in participants from Thailand and Sri Lanka, where JE vaccination is routine [6]. We report a detailed evaluation of the efficacy of TAK-003 from the first dose through 4.5 years after the second dose in participants with and without previous YF or JE vaccination, together with safety and immunogenicity data in these subpopulations.

## METHODS

### Trial Design and Participants

The results evaluated herein were obtained from participants in an ongoing, phase 3, double-blind, randomized, placebo-controlled trial (ClinicalTrials.gov NCT02747927) including healthy children and adolescents from 4 to 16 years of age at 26 sites in Brazil, Colombia, the Dominican Republic, Nicaragua, Panama, the Philippines, Sri Lanka, and Thailand. Informed assent or consent forms and the trial protocol and its amendments were approved by institutional review boards, independent ethics committees, and health authorities. Written informed assent or consent was obtained from participants, parents, or legal guardians before enrollment. Renewal of consent is obtained from participants as necessary with increasing age as the trial continues. The trial protocol was amended to include a booster evaluation phase in part of the initial trial population, which is ongoing. The data presented in this report cover the entire prebooster follow-up in the trial (ie, 4.5 years after second dose). The trial is following the ethical principles of the Declaration of Helsinki and the International Council for Harmonisation of Technical Requirements for Pharmaceuticals for Human Use harmonized tripartite guidelines for good clinical practice.

Details of the trial design, inclusion criteria, and exclusion criteria have been reported previously [6, 8, 10, 17]. In brief, 20 099 children were randomly assigned 2:1 to receive 2 doses of TAK-003 (n = 13 401) or 2 doses of placebo (n = 6698) 3 months apart; a subset of 4000 participants was randomly selected for additional safety and immunogenicity assessments. Data on the prior receipt of YF/JE vaccination was collected before the first trial vaccination and in follow-up visits until 1 month after the second trial vaccination. Subsequently, such data were collected only in the context of adverse event (AE) reporting. The collection of YF/JE vaccination history included supportive documentation (eg, a vaccination card). Further information on the baseline characteristics of the trial population is available in previous reports [6, 8, 10, 17].

### Trial Procedures

TAK-003 or placebo (saline solution) was administered subcutaneously into the upper arm at months 0 and 3. Participants were under active febrile surveillance for the trial duration to detect symptomatic dengue (both nonhospitalized and hospitalized), with weekly contacts and serotype-specific quantitative real-time polymerase chain reaction assay of acute samples (see Biswal et al [8] for full details). Blood samples to assay dengue neutralizing antibodies using a microneutralization test (MNT) were collected before vaccination from all participants at month 0 (day 1) and 1 month after the second dose (month 4). Additional assays were planned on days 30, 90, 270, and 450, and then annually to 4.5 years after the second dose from the subset of 4000 participants described earlier. MNT results are reported as the reciprocal of the serum dilution that resulted in a 50% reduction in plaque count compared with virus controls (MNT_50_). Serious AEs and AEs leading to trial withdrawal were monitored for the duration of the trial.

### Outcomes

Data on the primary and secondary objectives of the trial have been reported previously [6, 8, 10, 17]. Evaluation of the impact of prior JE or YF vaccination on VE, safety, and immunogenicity was exploratory and included both prespecified and post hoc analyses. The participants included in this analysis had been followed for approximately 4.5 years (54 months) since the second dose (57 months since the first dose). Cumulative VE was assessed from the first dose onward. Safety data for 28 days after any trial vaccination (ie, first or second dose) are reported in this analysis.

### Statistical Analysis

Vaccine efficacy was calculated as 1 – (λv/λc), where λv and λc are the hazard rates for the TAK-003 and placebo arms, respectively. Hazard ratios with 95% CIs were estimated using a Cox proportional hazard model with trial group as a factor, adjusted for age, and stratified by region; subjects were administratively censored at the time of the analysis data cut or at the time of last contact if lost to follow-up/discontinued. Cumulative VE was assessed in the safety set, which included all randomized participants who received at least 1 dose of TAK-003 or placebo and was summarized by investigational product and receipt of JE or YF vaccination before the first trial vaccination. Immunogenicity data were analyzed in the per-protocol set for immunogenicity, which included participants who had received at least 1 dose of TAK-003 or placebo, had at least 1 valid postdose blood sample, and had no major protocol violations. In this analysis, participants were stratified by YF and JE vaccination history. Full details of the statistical analysis and methods have been published previously [6, 8, 10, 17].

## RESULTS

### Participants

A total of 20 071 participants received at least 1 dose of TAK-003 or placebo and were included in the safety set. Participant characteristics have previously been reported [6, 8]. As shown in Table 1, 23.9% of the participants had been vaccinated against JE, including >90% of those in Sri Lanka and Thailand, and 21.1% had been vaccinated against YF, including 81.7% in Colombia and 49.1% in Brazil. Within the trial population and each country or region, the percentages of placebo and TAK-003 participants were balanced. As expected, JE vaccination was reported exclusively in Asia and YF vaccination exclusively in Latin America, except for 1 participant in Asia with a prior YF vaccination. As shown in Table 2, 78.8% in the YF subgroup, 63.8% in the JE subgroup, and 73.6% in the no YF/JE subgroup were seropositive at baseline, with MNT titers of at least 10 at randomization to 1 or more DENV serotypes. Within each subgroup, the seropositivity rates in the TAK-003 and placebo arms were similar.

**Table 1.** Prior or Concomitant Yellow Fever and Japanese Encephalitis Vaccination in Participants Who Received at Least 1 Dose of Trial Vaccine or Placebo (Safety Set Data)

Vaccine	Characteristic	No. (%) of Participants		
		Placebo (n = 6687)	TAK-003 (n = 13 380)	Total^a^ (n = 20 071)
Prior to trial				
JE	Overall	1579 (23.6)	3206 (24.0)	4787 (23.9)
	Supported by vaccination card or other documentation	1220 (18.2)	2489 (18.6)	3711 (18.5)
	Philippines	9/1327 (0.7)	45/2599 (1.7)	54/3926 (1.4)
	Sri Lanka	676/700 (96.6)	1327/1394 (95.2)	2005/2096 (95.7)
	Thailand	894/966 (92.5)	1834/2003 (91.6)	2728/2969 (91.9)
YF	Overall	1415 (21.2)	2822 (21.1)	4238 (21.1)
	Supported by vaccination card or other documentation	1307 (19.5)	2635 (19.7)	3943 (19.6)
	Brazil	262/560 (46.8)	607/1212 (50.1)	870/1773 (49.1)
	Colombia	1088/1319 (82.5)	2084/2564 (81.3)	3172/3884 (81.7)
	Panama	65/989 (6.6)	130/2010 (6.5)	195/2999 (6.5)
	Sri Lanka	0/700 (0.0)	1/1394 (<0.1)	1/2096 (<0.1)
During trial (from first dose to 54 mo after the second dose)^b^				
JE	Overall	0 (0.0)	4 (<0.1)	4 (<0.1)
YF	Overall	120 (1.8)	246 (1.8)	366 (1.8)

Data on receipt of concomitant YF or JE vaccination were collected from all participants in follow-up visits until 1 month after the second dose. Subsequently, data were only collected in the context of adverse event evaluation. The denominator for the calculation of percentages is the number of participants in the subgroup.

Abbreviations: JE, Japanese encephalitis; YF, yellow fever.

^a^Total includes 4 participants who received both TAK-003 and placebo because of an administrative error and, hence, were excluded from the TAK-003 and placebo groups.

^b^JE and YF vaccine administration data were collected until the month 4 visit (ie, 1 month after the second dose).

**Table 2.** Baseline Dengue Seropositivity of Participants With Prior Japanese Encephalitis or Yellow Fever Vaccination (Safety Set Data)

Prior/Concurrent Vaccination	No. Evaluated	Baseline Dengue Seropositivity (MNT >10), No. (%) of Participants						
		≥1 Serotype	DENV-1	DENV-2	DENV-3	DENV-4	At Least Trivalent	Tetravalent
JE								
Placebo (n = 1579)	1579	983 (62.3)	770 (48.8)	941 (59.6)	756 (47.9)	788 (49.9)	754 (47.8)	715 (45.3)
TAK-003 (n = 3206)	3206^a^	2070 (64.6)	1650 (51.5)	1991 (62.1)	1619 (50.5)	1683 (52.5)	1619 (50.5)	1538 (48.0)
Total (n = 4787)	4787^b^	3054 (63.8)	2420 (50.6)	2933 (61.3)	2375 (49.6)	2471 (51.6)	2373 (49.6)	2253 (47.1)
YF								
Placebo (n = 1414)	1414	1133 (80.1)	1045 (73.9)	1112 (78.6)	996 (70.4)	993 (70.2)	1002 (70.9)	948 (67.0)
TAK-003 (n = 2822)	2821	2205 (78.2)	2069 (73.3)	2164 (76.7)	1946 (69.0)	1945 (68.9)	1970 (69.8)	1870 (66.3)
Total (n = 4237)	4236	3339 (78.8)	3115 (73.5)	3277 (77.4)	2943 (69.5)	2939 (69.4)	2973 (70.2)	2819 (66.5)
No YF/JE								
Placebo (n = 3694)	3693	2738 (74.1)	2521 (68.3)	2661 (72.1)	2437 (66.0)	2452 (66.4)	2442 (66.1)	2340 (63.4)
TAK-003 (n = 7353)	7351	5389 (73.3)	4987 (67.8)	5253 (71.5)	4854 (66.0)	4900 (66.7)	4866 (66.2)	4682 (63.7)
Total (n = 11 048)	11 045	8128 (73.6)	7509 (68.0)	7915 (71.7)	7292 (66.0)	7353 (66.6)	7309 (66.2)	7023 (63.6)

Abbreviations: DENV, dengue virus; JE, Japanese encephalitis; MNT, microneutralization test; YF, yellow fever.

^a^3204 participants evaluated for DENV-2, DENV-3, at least trivalent, and tetravalent.

^b^4785 participants evaluated for DENV-2, DENV-3, at least trivalent, and tetravalent.

### Background Virologically Confirmed Dengue Distribution (Safety Set Data in Placebo Group)

During approximately 57 months of follow-up, there were 560 cases of VCD in the placebo group (safety set), including 13 cases of sequential episodes of VCD [21]; 142 of those cases (∼25%) required hospitalization. All 4 serotypes were reported from Asian sites, whereas VCD cases at Latin American sites were predominantly caused by DENV-1 or DENV-2.

**Figure 1.**
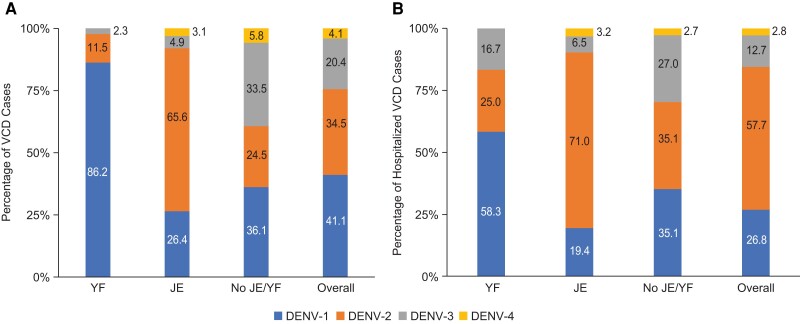
Serotype distribution of virologically confirmed dengue (VCD) (*A*) and hospitalized VCD (*B*) in placebo group participants with and without prior yellow fever or Japanese encephalitis vaccination (safety set data from first vaccination to approximately 57 mo after first dose or 54 mo after second dose). Serotype distribution data include the instances of second episodes of VCD in 13 participants after initiating trial vaccination. Abbreviations: DENV, dengue virus; JE, Japanese encephalitis; VCD, virologically confirmed dengue; YF, yellow fever.

Seventy-five of the 87 cases of VCD that occurred in the YF vaccination subgroup (86.2%) were caused by DENV-1, and 107 of the 163 cases in the JE vaccination subgroup (65.6%) were caused by DENV-2. Of the 310 cases of VCD that occurred in participants without previous YF/JE vaccination, 112 (36.1%) were caused by DENV-1, 76 (24.5%) by DENV-2, and 104 (33.5%) by DENV-3 (Figure 1*A*). Trends in the serotype distribution of cases of hospitalized VCD were generally similar to those seen for VCD overall (Figure 1*B*).

**Figure 2.**
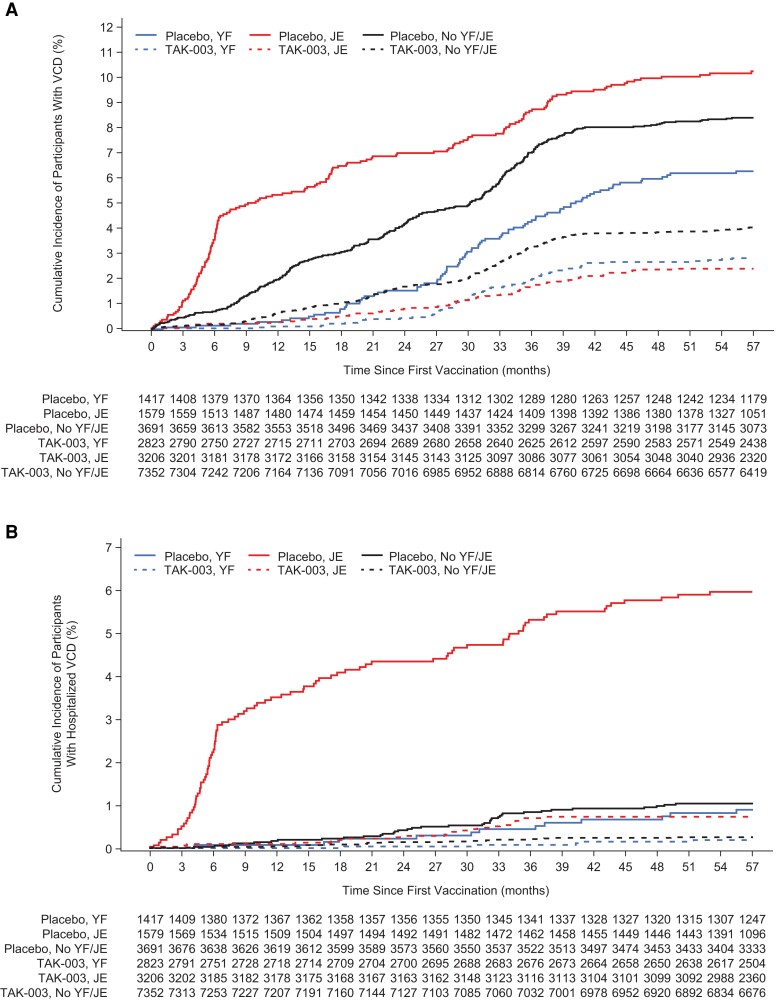
Cumulative incidence of virologically confirmed dengue (VCD) (*A*) and hospitalized VCD (*B*) over 57 mo after first vaccination in participants with or without prior vaccination against yellow fever or Japanese encephalitis (safety set data). Abbreviations: JE, Japanese encephalitis; VCD, virologically confirmed dengue; YF, yellow fever.

The cumulative incidence of VCD in the placebo participants revealed differing disease burdens and case-occurrence patterns in the 3 subgroups by prior YF/JE vaccination overall (Figure 2) and by serotype (Supplementary Figure 1).

### VE Against Virologically Confirmed Dengue and Hospitalized Virologically Confirmed Dengue in the Subgroups Over Approximately 57 Months of Follow-up (Safety Set Data)

**Figure 3.**
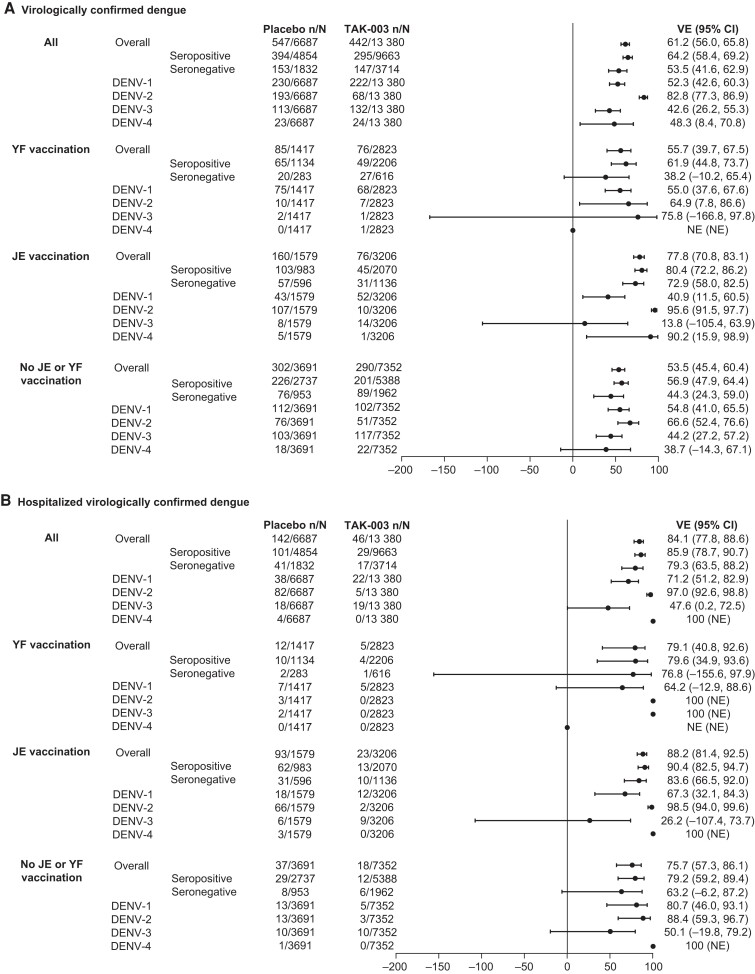
Vaccine efficacy (VE) (95% confidence interval) in preventing virologically confirmed dengue (VCD) (*A*) and hospitalized VCD (*B*) from the first vaccine dose until 4.5 years after the second dose (approximately 57 mo) in participants with and without prior yellow fever or Japanese encephalitis vaccination (safety set data). Percentages were calculated from the number of participants who were evaluated for VCD. Only the first VCD case in a participant was included in the overall VE analysis. VE analysis by serotype included the first VCD case for a specific serotype in an individual participant. Participants were classified as seronegative if they tested seronegative for all dengue serotypes at baseline. Participants were classified as seropositive if they demonstrated a reciprocal neutralizing antibody titer ≥10 against at least 1 dengue virus serotype at baseline. Abbreviations: CI, confidence interval; DENV, dengue virus; JE, Japanese encephalitis; NE, not evaluable; VE, vaccine efficacy; YF, yellow fever.

The number of cases of VCD and VE by prior YF/JE vaccination are shown in Figure 3*A*. The overall VE (95% CI) against VCD was 55.7% (39.7%–67.5%) in those with YF vaccination, compared with 77.8% (70.8%–83.1%) in those with JE vaccination, and 53.5% (45.4%–60.4%) in those with no YF/JE vaccination. VE (95% CI) against hospitalized VCD was high, irrespective of prior YF/JE vaccination, and was 79.1% (40.8%–92.6%) in the YF vaccination subgroup, 88.2% (81.4%–92.5%) in the JE vaccination subgroup, and 75.7% (57.3%–86.1%) in the no YF/JE vaccination subgroup (Figure 3*B*).

In baseline seropositive participants, VE (95% CI) was 61.9% (44.8%–73.7%) in the YF vaccination subgroup, 80.4% (72.2%–86.2%) in the JE vaccination subgroup, and 56.9% (47.9%–64.4%) in the no YF/JE vaccination subgroup. In baseline seronegative participants, VE was 38.2% (−10.2% to 65.4%) in the YF vaccination subgroup, 72.9% (58.0%–82.5%) in the JE vaccination subgroup, and 44.3% (24.3%–59.0%) in the no YF/JE vaccination subgroup (Figure 3*A*). The corresponding VE point estimates against hospitalized VCD were numerically higher in the different subgroups according to prior YF/JE vaccination and baseline serostatus, although certain estimates were based on a very small number of cases (Figure 3*B*).

### VE Against Virologically Confirmed Dengue and Hospitalized Virologically Confirmed Dengue by Serotype in the Subgroups Over Approximately 57 Months of Follow-up (Safety Set Data)

Data were further explored to understand the impact of prior YF/JE vaccination on performance of the vaccine at serotype level. For DENV-1, VE (95% CI) was 55.0% (37.6%–67.6%) in the YF vaccination subgroup, 40.9% (11.5%–60.5%) in the JE vaccination subgroup, and 54.8% (41.0%–65.5%) in the no YF/JE vaccination subgroup. The corresponding VE (95% CI) against hospitalized VCD was 64.2% (−12.9% to 88.6%), 67.3% (32.1%–84.3%), and 80.7% (46.0%–93.1%), respectively. For DENV-2, VE (95% CI) was 64.9% (7.8%–86.6%) in the YF vaccination subgroup, 95.6% (91.5%–97.7%) in the JE vaccination subgroup, and 66.6% (52.4%–76.6%) in the no YF/JE vaccination subgroup. VE against hospitalized dengue was high across the 3 subgroups and ranged from 88.4% (95% CI, 59.3%–96.7%) to 100% (not evaluable [NE]).

Such analysis for DENV-3 and DENV-4 was limited by sparse case incidence in the YF vaccination and JE vaccination subgroups.

The overall VE (95% CI) against VCD for DENV-1, DENV-2, DENV-3, and DENV-4 was 52.3% (42.6%–60.3%), 82.8% (77.3%–86.9%), 42.6% (26.2%–55.3%), and 48.3% (8.4%–70.8%), respectively (Figure 3*A*). The overall VE (95% CI) against hospitalized VCD for DENV-1, DENV-2, DENV-3, and DENV-4 was 71.2% (51.2%–82.9%), 97.0% (92.6%–98.8%), 47.6% (0.2%–72.5%), and 100% (NE), respectively (Figure 3*B*).

### Serious Adverse Events Within 28 Days of Vaccination

Serious AEs were experienced by 23 of 4241 YF vaccination subgroup participants and 46 of 4787 JE vaccination subgroup participants in the 28 days after any trial vaccination. No deaths occurred during that time (Table 3). One participant each in the YF and JE vaccination subgroups had serious AEs (hypersensitivity and dengue hemorrhagic fever, respectively) that the investigator judged to be related to the blinded investigational product; both were in the placebo group. Serious AEs led to investigational product withdrawal by 1 TAK-003 recipient (seizure) and 1 placebo recipient (hypersensitivity) in the YF vaccination subgroup. The case of hypersensitivity judged to be related to the blinded investigational product also led to investigational product withdrawal.

**Table 3.** Participants With Serious Adverse Events up to 28 Days by System Organ Class After Any Vaccination (Safety Set Data)

Serious Adverse Event	Prior YF Vaccine, No. (%) of Participants		Prior JE Vaccine, No. (%) of Participants	
	TAK-003	Placebo	TAK-003	Placebo
	(n = 2823)	(n = 1417)	(n = 3206)	(n = 1579)
Related to investigational product^a^	0	1 (<0.1)	0	1 (<0.1)
Immune system disorders	0	1 (<0.1)	0	0
Infections and infestations	0	0	0	1 (<0.1)
Not related to investigational product	13 (0.5)	9 (0.6)	29 (0.9)	16 (1.0)
Blood and lymphatic system disorders	1 (<0.1)	0	1 (<0.1)	0
Eye disorders	0	0	1 (<0.1)	0
Gastrointestinal disorders	1 (<0.1)	0	2 (<0.1)	0
General disorders and administration site conditions	0	0	1 (<0.1)	0
Immune system disorders	0	0	1 (<0.1)	0
Infections and infestations	3 (0.1)	2 (0.1)	21 (0.7)	9 (0.6)
Injury, poisoning, and procedural complications	3 (0.1)	4 (0.3)	2 (<0.1)	6 (0.4)
Nervous system disorders	3 (0.1)	0	1 (<0.1)	0
Psychiatric disorders	1 (<0.1)	0	0	1 (<0.1)
Renal and urinary disorders	1 (<0.1)	0	0	0
Respiratory, thoracic, and mediastinal disorders	1 (<0.1)	1 (<0.1)	0	0
Skin and subcutaneous tissue disorders	0	0	1 (<0.1)	0
Social circumstances	0	2 (0.1)	0	0
Leading to withdrawal of investigational product	1 (<0.1)	1 (<0.1)	0	0
Immune system disorders	0	1 (<0.1)	0	0
Nervous system disorders	1 (<0.1)	0	0	0
Deaths	0	0	0	0

Abbreviations: JE, Japanese encephalitis; YF, yellow fever.

^a^As assessed by investigator. Percentages are based on the number of participants who received the specific vaccination within each trial group. Participants with 1 or more AEs are counted only once using the most related/most severe event.

### Immunogenicity

**Figure 4.**
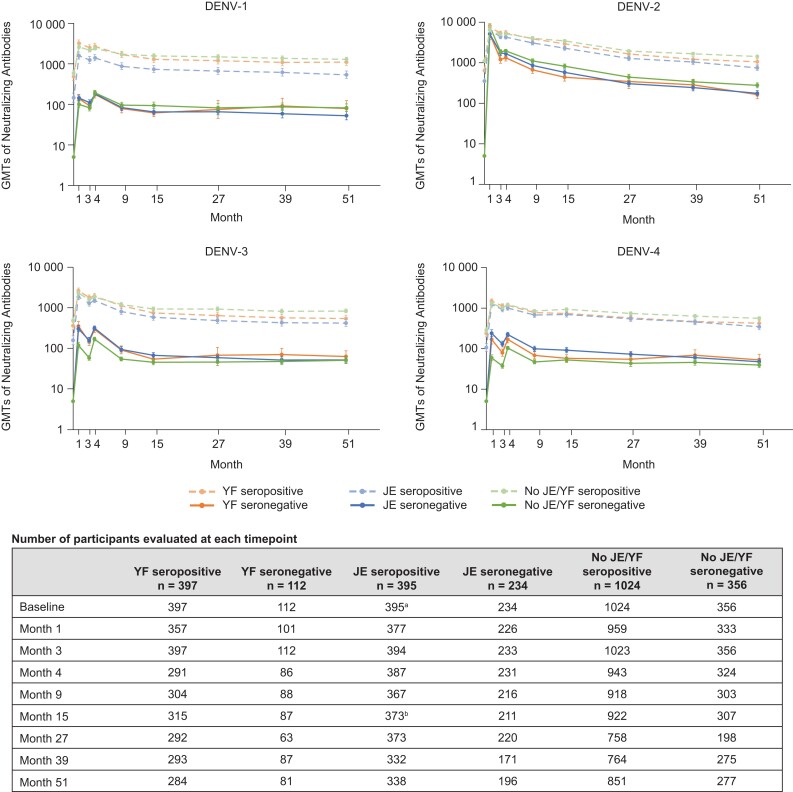
Geometric mean titers (with 95% confidence interval) of neutralizing antibodies measured by microneutralization test for each dengue virus (DENV) serotype in baseline seropositive and baseline seronegative participants with and without prior yellow fever or Japanese encephalitis vaccination (per-protocol set immunogenicity data). Number of participants evaluated at each timepoint may vary. Unless noted, the number of participants evaluated at each timepoint were the same for all serotypes. ^a^For DENV-2 and DENV-3, n = 394. ^b^For DENV-1, n = 371; for DENV-2, n = 372. Abbreviations: DENV, dengue virus; GMT, geometric mean titer; JE, Japanese encephalitis; YF, yellow fever.

Geometric mean titers (GMTs) of neutralizing antibodies for each dengue serotype in baseline seropositive and baseline seronegative participants are shown in Figure 4. Generally, GMTs were of similar magnitude in the baseline seronegative participants, irrespective of prior YF or JE vaccination, without any consistent pattern of difference across the 4 serotypes between the 3 subgroups. In the JE seronegative and YF seronegative group, GMTs reached a higher peak compared to the no JE/YF seronegative group for DENV-3 and DENV-4. Tetravalent seropositivity was reached for >99% of participants. Seropositivity rates (MNT ≥10) against each serotype remained high (mostly >90%) following vaccination through the end of the follow-up period, with no clear differences observed, irrespective of prior JE or YF vaccination (Supplementary Figure 2).

## DISCUSSION

There is biological plausibility for prior flavivirus vaccination to have an impact on subsequent dengue infection and, hence, potentially on the performance of a live-attenuated dengue vaccine [22–24]. The wide geographic setting of this efficacy trial provided an opportunity to assess that impact. JE vaccination coverage was >90% in the trial populations in both Sri Lanka and Thailand, while YF vaccination coverage was >80% in the trial population in Colombia and 49% in Brazil. Overall, these participants with a prior YF/JE vaccination accounted for approximately 45% of the trial population. The remaining approximately 55% of the trial population without a prior YF/JE vaccination was mostly in the Philippines, Panama, Dominican Republic, and Nicaragua, representing a potential control subgroup (ie, no YF/JE vaccination). We planned to collect YF/JE vaccination history, and the vast majority of participants had supporting documentation.

Fifty-seven months after initiating vaccination, the overall VE for TAK-003 against symptomatic dengue was 55.7% in those with prior YF, 77.8% in those with prior JE, and 53.5% in those without prior YF/JE vaccination. These values would suggest better performance of TAK-003 in recipients with prior JE vaccination. To some extent, this pattern was also reflected in VE against hospitalized VCD, with efficacy of 79.1% in those with prior YF, 88.2% in those with prior JE, and 75.7% in those without prior YF/JE vaccination. However, the apparent difference can be explained by geographical differences in the background circulating serotypes, as observed in the corresponding placebo groups, differences in JE and YF vaccination rates across the countries included in the trial, and the varied performance of TAK-003 against individual serotypes—a trend of VE against DENV-2 > DENV-1 > DENV-3 [6]. Another consideration is difference in time of dengue case occurrence among the subgroups due to epidemiological factors along with some waning in efficacy over time [10, 17].

In placebo recipients with prior JE vaccination, 65.6% of VCD cases were caused by DENV-2 (against which TAK-003 VE was highest) versus 11.5% and 24.5% in the YF vaccination subgroup and no YF/JE vaccination subgroup, respectively. Further exploration at the serotype level suggested higher efficacy against DENV-2 VCD in the prior JE vaccination subgroup (VE of 95.6% vs 64.9% in the YF vaccination subgroup and 66.6% in the no YF/JE vaccination subgroup). This is likely confounded by differential case acquisition time in the 3 subgroups and the VE over time. In the JE vaccination subgroup, most of the case incidence occurred soon after vaccination. Among placebo recipients, 75.7% (81/107) of DENV-2 VCD cases in the JE vaccination subgroup occurred from the first dose until 1 year after the second dose (∼15 months); in this timeframe, 20.0% (2/10) of cases in the YF vaccination subgroup and 11.8% (9/76) of cases in the no YF/JE vaccination subgroup were reported. This is also evident in the serotype-level cumulative incidence curve, reflecting the major DENV-2 outbreak in Sri Lanka in 2017. During the first year after vaccination, VE (95% CI) against DENV-2 was 97.7% (92.7%–99.3%), which declined to 75.0% (52.3%–86.9%) during year 2, remained at 76.4% (61.8%–85.4%) during year 3, and declined to 72.8% (7.2%–92.1%) during year 4 after vaccination.

At baseline, 79% of the YF vaccination participants, 64% of the JE vaccination participants, and 74% of no YF/JE vaccination participants were seropositive, with MNT titers to 1 or more dengue serotypes of at least 10, likely reflecting regional differences in seroprevalence. This shows that a substantial proportion of participants were seronegative per the dengue neutralization assay after vaccination against flavivirus. The pattern and magnitude of antibody titers to TAK-003 vaccination against each of the 4 serotypes were broadly similar across the 3 subgroups according to YF/JE vaccination status. Any apparent differences in immune response patterns among the 3 subgroups were not consistent across the 4 serotypes. Likewise, the seropositivity rates against individual serotypes and multivalent serotypes were high and similar after the 2-dose TAK-003 vaccination regimen across the 3 subgroups.

The safety data did not suggest any clinically important differences between TAK-003 and placebo, regardless of prior YF/JE vaccination. In this ongoing trial, the vaccine safety profile remained favorable during 4.5 years of postvaccination follow-up [18].

Interestingly, immunogenicity data from the present analysis are consistent with findings from another study in which effects of concomitant and sequential administration of YF-17D and TAK-003 vaccines on immunogenicity, among other endpoints, were assessed in healthy adults aged 18–60 years living in the United States [25]. This study showed no reduction in immune response to TAK-003 or YF-17D when the vaccines were administered concomitantly. Compared to concomitant administration, higher DENV GMTs were observed following sequential vaccination, which may suggest an enhancing effect of YF-17D on TAK-003 antibody responses when YF-17D is administered before or after the TAK-003 vaccine. However, without immune correlates of protection for dengue, the clinical significance of this potential effect is unknown.

The cumulative VCD incidence overall and at serotype level in the placebo group showed differences in disease burden and regional dengue epidemiology. The greatest disease burden was in the JE vaccination subgroup (regions in Asia), with a rapid increase in VCD in the first 6 months that was driven by a dengue outbreak in Sri Lanka in 2017. The YF vaccination subgroup (regions in Latin America) had the lowest dengue burden, but the incidence curve showed an increasing trend after the first year postvaccination. The cumulative incidence curves flattened to some extent in the last 18 months alongside the lower dengue incidence during the COVID-19 pandemic. The serotype distribution also varied considerably between the YF/JE prior vaccination subgroups, reflecting circulating serotypes in the regions.

Several limitations of this analysis should be noted. First, the trial was neither designed for subgroup comparisons by prior YF or JE vaccination, nor were the participants randomized accordingly, and the differing dengue epidemiology was a considerable confounding factor in data interpretation. Second, for epidemiological reasons, all dengue serotypes were not equally represented in VE analysis. Third, additional participants might have received YF or JE vaccines during the trial, but this could not be considered in the analysis because it was not planned to systematically collect that data following the first month after the second vaccination. Additionally, YF and JE vaccination was confounded by region/country owing to different vaccination policies and disease endemicity. Finally, interpretation of the serotype-specific results was limited by small DENV-3 and DENV-4 case counts in certain subgroups.

In summary, despite the limitations acknowledged, the available data do not suggest a clinically meaningful impact of prior JE or YF vaccination on TAK-003 performance. Overall, TAK-003 was well-tolerated and efficacious in different epidemiological settings, irrespective of prior YF/JE vaccination.

## Supplementary Data

Supplementary materials are available at *The Journal of Infectious Diseases* online (http://jid.oxfordjournals.org/). Supplementary materials consist of data provided by the author that are published to benefit the reader. The posted materials are not copyedited. The contents of all supplementary data are the sole responsibility of the authors. Questions or messages regarding errors should be addressed to the author.

## Supplementary Material

jiae222_Supplementary_Data

## Notes

***Acknowledgments.*** The authors sincerely thank the trial participants and their parents/guardians who agreed to participate in the trial; the data monitoring committee; the Dengue Case Adjudication Committee members; the Takeda expanded trial team; the study team at PPD; and all the trial staff in each of the countries. The authors also acknowledge the contribution of the late Dan Stinchcomb, PhD, and Inviragen Inc (Fort Collins, Colorado) in the initial developmental work on TAK-003 (Inviragen Inc was subsequently acquired by Takeda). Medical writing support was provided by Clement Weinberger, PhD, of The Stylus, and Jenny Engelmoer, PhD, of Sula Communications, and funded by Takeda. Editorial support was provided by Excel Medical Affairs, funded by Takeda.

***Author contributions.*** S. B., M. R., A. B., and D. W. designed the trial. S. B., M. R., A. B., I. L., I. E., S. T., E. L., H. F., V. T., N. F., and D. W. were involved in data analysis and interpretation. S. B., V. T., and I. E. managed the trial. All other authors were trial investigators.

***Data sharing.*** The datasets, including the redacted study protocol, redacted statistical analysis plan, and individual participants’ data supporting the results of the completed study, will be made available within 3 months from initial request to researchers who provide a methodologically sound proposal. The data will be provided after de-identification, in compliance with applicable privacy laws, data protection, and requirements for consent and anonymization. Data requests should follow the process described in the data sharing section on https://clinicaltrials.takeda.com/ and https://vivli.org/ourmember/takeda/.

***Financial support*.** This analysis was funded by Takeda.

## APPENDIX

***TIDES study group investigators.*** Asvini Fernando, MD, Faculty of Medicine, University of Kelaniya, Sri Lanka; Charissa Borja-Tabora, MD, Research Institute for Tropical Medicine, Muntinlupa, Philippines; Chukiat Sirivichayakul, MD, Department of Tropical Pediatrics, Faculty of Tropical Medicine, Mahidol University, Thailand; Delia Yu, MD, De La Salle Health Sciences Institute, Dasmariñas, Philippines; Dulanie Gunasekera, MD, FRCP, Faculty of Medical Sciences, University of Sri Jayawardenenpura, Sri Lanka; Eduardo López-Medina, MD, Centro de Estudios en Infectologia Pediátrica, Universidad del Valle and Centro Médico Imbanaco, Cali, Colombia; Edith Johanna Rodriguez-Arenales, MD, Centro de Atención e Investigación Médica (CAIMED), Aguazul, Colombia; Edson Duarte Moreira Jr, MD, Associação Obras Sociais Irmã Dulce Hospital Santo Antônio and Oswaldo Cruz Foundation, Bahia, Brazil; Felix Espinoza, MD, National Autonomous University of Nicaragua, León, Nicaragua; Hector Velásquez, MD, CAIMED, Acacias, Colombia; Humberto Reynales, MD, CAIMED, Bogotá, Colombia; Kleber Luz, MD, Instituto de Medicina Tropical da Universidade Federal do Rio Grande do Norte, Brazil; Jose Jimeno, MD, Rodrigo deAntonio, MD, and Elaine Aranguren, MD, Centro de Vacunación Internacional (Cevaxin), Panama City, Panama; LakKumar Fernando, MD, FRCP, Centre for Clinical Management of Dengue and Dengue Haemorrhagic Fever, Negombo General Hospital, Negombo, Sri Lanka; Lulu Bravo, MD, University of the Philippines Manila, Ermita, Philippines; Luis Martinez Vargas, MD, CAIMED, Dominicana, Santo Domingo, Dominican Republic; Luis Rivera, MD, Hospital Maternidad Nuestra Senora de Altagracia, Santo Domingo, Dominican Republic; Maria Theresa Alera, MD, Philippines-AFRIMS Virology Research Unit, Cebu, Philippines; Onanong Manacharoen, MD, Department of Pediatrics, Buddhasothorn Hospital, Thailand; Pio Lopez, MD, Centro de Estudios en Infectologia Pediátrica, Universidad del Valle, Cali, Colombia; Pope Kosalaraksa, MD, Faculty of Medicine, Khon Kaen University, Khon Kaen, Thailand; V. Pujitha Wickramasinghe, MD, PhD, University of Colombo, Sri Lanka; Reynaldo Dietze, MD, Núcleo de Doenças Infecciosas, Centro de Ciências da Saúde, Universidade Federal do Espirito Santo, Vitória, Brazil; Rivaldo Venâncio da Cunha, MD, Fundação Oswaldo Cruz (FIOCRUZ), Universidade Federal de Mato Grosso do Sul, Campo Grande, Brazil; Veerachai Watanaveeradej, MD, Phramongkutklao Hospital, Bangkok, Thailand; Xavier Saez-Llorens, MD, Hospital del Niño Dr José Renán Esquivel, Sistema Nacional de Investigación at SENACYT, Centro de Vacunación Internacional (Cevaxin), Panama City, Panama; Manja Brose, MSc, Martina Rauscher, PhD, Olaf Zent, MD, Inge LeFevre, MD, Astrid Borkowski, MD, PhD, Ivo Sonderegger, PhD, and Vianney Tricou, PharmD, DPhil, Takeda Pharmaceuticals International AG, Zurich, Switzerland; Shibadas Biswal, MD, Mengya Liu, PhD, Elaine Hoffman, PhD, Ian Escudero, MD, and Derek Wallace, MBBS, Takeda Vaccines, Inc, Boston, Massachusetts; Yanee Hutagalung, MD, Takeda Vaccines Pte Ltd, Singapore; Suely Tuboi, MD, Takeda Pharmaceuticals Ltda, Sao Paulo, Brazil.
