# Supplementary material for: Efficacy and Safety of a Tetravalent Dengue Vaccine (TAK-003) in Children With Prior Japanese Encephalitis or Yellow Fever Vaccination
Source: J Infect Dis. 2024 Apr 29;230(6):e1214–25. doi: 10.1093/infdis/jiae222 (PMC11646590; doi:10.1093/infdis/jiae222)
Supplement: jiae222_Supplementary_Data [file jiae222_supplementary_data.zip › Tricou_SupplementaryMaterials_FigureS1.pdf]

Supplementary Figure 1. Cumulative incidence of virologically confirmed dengue (VCD) caused by (A) dengue virus (DENV)-1, (B) DENV-2, (C) DENV-3, and (D) DENV-4 over 57 months after first vaccination in participants with or without prior vaccination against yellow fever (YF) or Japanese encephalitis (JE) (safety set data).

A DENV-1

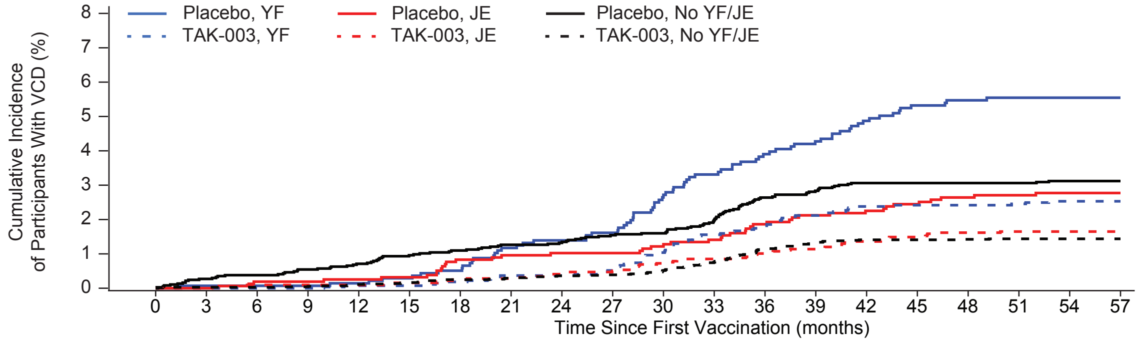

	0	3	6	9	12	15	18	21	24	27	30	33	36	39	42	45	48	51	54	57
Placebo, YF	1417	1408	1380	1372	1366	1358	1352	1344	1340	1337	1316	1306	1294	1287	1270	1264	1255	1251	1243	1188
Placebo, JE	1579	1576	1566	1562	1560	1558	1548	1546	1544	1544	1536	1523	1515	1510	1505	1500	1494	1492	1437	1118
Placebo, No YF/JE	3691	3666	3625	3610	3599	3584	3567	3553	3540	3522	3511	3492	3457	3443	3420	3397	3379	3362	3331	3260
TAK-003, YF	2823	2791	2751	2728	2716	2712	2704	2695	2691	2682	2661	2643	2629	2618	2604	2597	2590	2577	2555	2445
TAK-003, JE	3206	3203	3185	3182	3178	3174	3166	3165	3157	3155	3139	3113	3107	3101	3085	3078	3072	3064	2959	2334
TAK-003, No YF/JE	7352	7312	7253	7226	7202	7185	7147	7130	7109	7083	7057	7019	6964	6924	6894	6868	6835	6808	6752	6599

B DENV-2

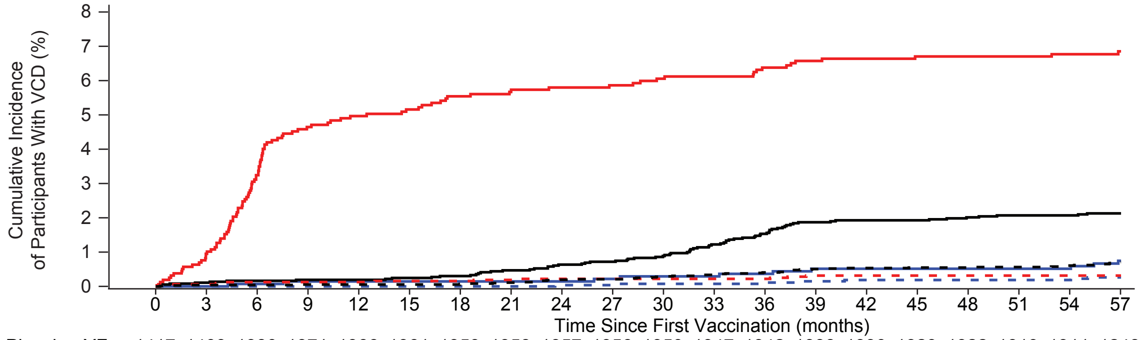

	0	3	6	9	12	15	18	21	24	27	30	33	36	39	42	45	48	51	54	57
Placebo, YF	1417	1409	1380	1371	1366	1361	1359	1358	1357	1356	1350	1347	1342	1338	1330	1329	1322	1319	1311	1248
Placebo, JE	1579	1561	1518	1492	1486	1482	1474	1472	1469	1468	1460	1449	1444	1440	1436	1433	1430	1429	1379	1093
Placebo, No YF/JE	3691	3672	3633	3623	3618	3610	3596	3582	3565	3552	3537	3519	3497	3478	3460	3437	3416	3396	3367	3294
TAK-003, YF	2823	2791	2751	2728	2718	2714	2709	2705	2700	2695	2687	2683	2675	2671	2663	2657	2649	2637	2617	2503
TAK-003, JE	3206	3201	3184	3181	3177	3174	3169	3168	3164	3164	3154	3132	3131	3126	3117	3114	3112	3105	3000	2367
TAK-003, No YF/JE	7352	7311	7252	7225	7206	7192	7160	7144	7121	7096	7074	7048	7016	6980	6955	6928	6895	6868	6807	6642

C DENV-3

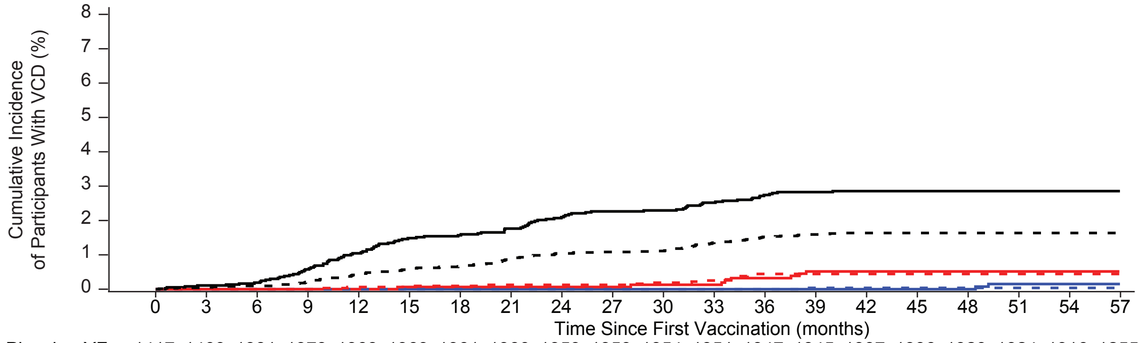

	0	3	6	9	12	15	18	21	24	27	30	33	36	39	42	45	48	51	54	57
Placebo, YF	1417	1409	1381	1373	1368	1363	1361	1360	1359	1359	1354	1351	1347	1345	1337	1336	1329	1324	1316	1255
Placebo, JE	1579	1576	1569	1565	1564	1562	1560	1560	1559	1559	1553	1544	1540	1536	1533	1531	1528	1527	1473	1148
Placebo, No YF/JE	3691	3672	3632	3609	3587	3565	3550	3535	3512	3496	3486	3472	3453	3443	3426	3404	3385	3367	3338	3266
TAK-003, YF	2823	2791	2751	2728	2718	2714	2709	2705	2701	2696	2689	2685	2678	2674	2667	2661	2653	2641	2621	2508
TAK-003, JE	3206	3203	3188	3185	3179	3176	3171	3170	3167	3167	3155	3131	3124	3122	3113	3110	3108	3101	2997	2367
TAK-003, No YF/JE	7352	7310	7248	7214	7179	7155	7118	7091	7063	7036	7017	6978	6939	6905	6879	6853	6821	6794	6737	6577

D DENV-4

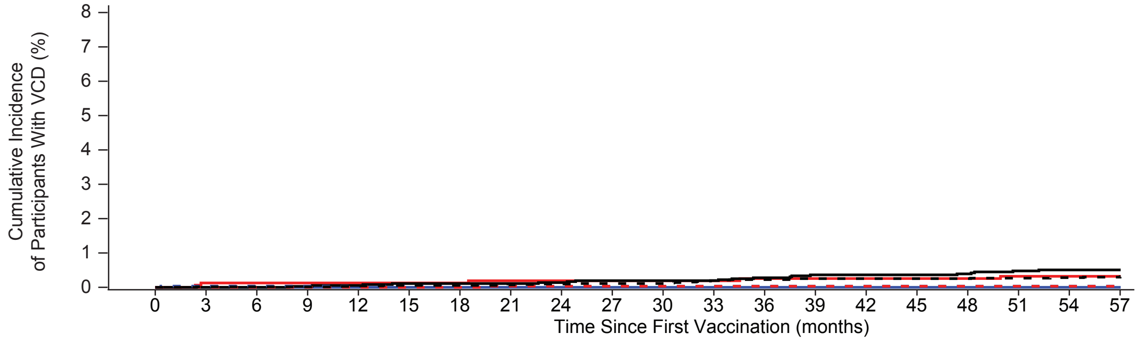

	0	3	6	9	12	15	18	21	24	27	30	33	36	39	42	45	48	51	54	57
Placebo, YF	1417	1409	1381	1373	1368	1363	1361	1360	1359	1359	1354	1351	1347	1345	1337	1336	1329	1326	1318	1257
Placebo, JE	1579	1574	1567	1563	1562	1561	1559	1558	1557	1557	1552	1542	1540	1539	1536	1534	1531	1529	1475	1149
Placebo, No YF/JE	3691	3676	3639	3629	3623	3615	3603	3595	3583	3571	3562	3556	3542	3532	3516	3493	3473	3452	3422	3350
TAK-003, YF	2823	2790	2750	2727	2717	2713	2708	2704	2700	2695	2688	2684	2677	2674	2667	2661	2653	2641	2621	2508
TAK-003, JE	3206	3203	3188	3185	3181	3179	3174	3173	3170	3170	3160	3138	3137	3135	3126	3123	3121	3114	3009	2374
TAK-003, No YF/JE	7352	7313	7254	7228	7207	7192	7161	7147	7128	7103	7086	7058	7029	6998	6975	6949	6917	6889	6830	6669
