# Supplementary material for: Efficacy and Safety of a Tetravalent Dengue Vaccine (TAK-003) in Children With Prior Japanese Encephalitis or Yellow Fever Vaccination
Source: J Infect Dis. 2024 Apr 29;230(6):e1214–25. doi: 10.1093/infdis/jiae222 (PMC11646590; doi:10.1093/infdis/jiae222)
Supplement: jiae222_Supplementary_Data [file jiae222_supplementary_data.zip › Tricou_SupplementaryMaterials_FigureS2.pdf]

Supplementary Figure 2. Seropositivity rates for each dengue virus (DENV) serotype after the first TAK-003 vaccination in participants with and without prior or concurrent Japanese encephalitis (JE) or yellow fever (YF) vaccination and positive or negative DENV baseline serostatus (per protocol set immunogenicity data). Number of participants evaluated at each timepoint may vary. Percentages are based on the number of participants evaluated at each timepoint. Unless noted, the number of participants evaluated at each timepoint were the same for all serotypes. [a]For DENV-2, DENV-3, at least trivalent, and tetravalent, n=394. [b]For DENV-1 and tetravalent, n=371; for DENV-2 and at least trivalent, n=372.

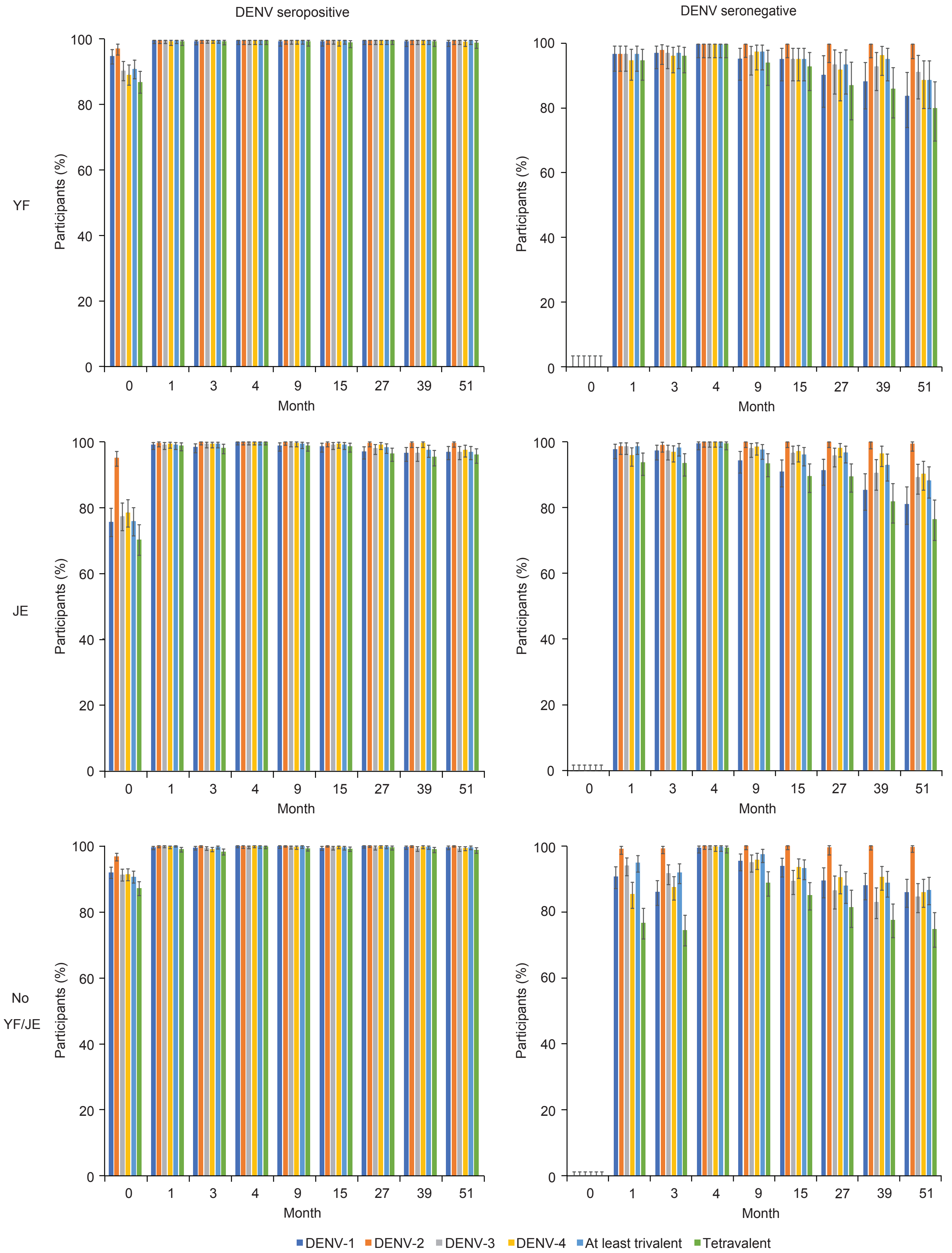

Number of participants evaluated at each timepoint

	YF seropositive n = 397	YF seronegative n = 112	JE seropositive n = 395	JE seronegative n = 234	No JE/YF seropositive n = 1024	No JE/YF seronegative n = 356
Baseline	397	112	395[a]	234	1024	356
Month 1	357	101	377	226	959	333
Month 3	397	112	394	233	1023	356
Month 4	291	86	387	231	943	324
Month 9	304	88	367	216	918	303
Month 15	315	87	373[b]	211	922	307
Month 27	292	63	373	220	758	198
Month 39	293	87	332	171	764	275
Month 51	284	81	338	196	851	277
